# The impact of COVID-19 on the hot food takeaway planning regulatory environment: perspectives of local authority professionals in the North East of England

**DOI:** 10.1177/17579139221106343

**Published:** 2022-08-05

**Authors:** HJ Moore, AA Lake, CL O’Malley, C Bradford, N Gray, M Chang, C Mathews, TG Townshend

**Affiliations:** School of Social Sciences, Humanities & Law, Teesside University, Middlesbrough, UK; Fuse, The Centre for Translational Research in Public Health, Newcastle upon Tyne, UK; Centre for Public Health Research, School of Health & Life Sciences, Teesside University, Middlesbrough TS1 3BA, UK; Fuse, The Centre for Translational Research in Public Health, Newcastle upon Tyne, UK; Centre for Public Health Research, School of Health & Life Sciences, Teesside University, Middlesbrough, UK; Fuse, The Centre for Translational Research in Public Health, Newcastle upon Tyne, UK; Centre for Public Health Research, School of Health & Life Sciences, Teesside University, Middlesbrough, UK; Fuse, The Centre for Translational Research in Public Health, Newcastle upon Tyne, UK; School of Social Sciences, Humanities & Law, Teesside University, Middlesbrough, UK; Healthy Places, Public Health England, London, UK WHO Collaborating Centre for Healthy Urban Environments, University of the West of England, Bristol, UK; Health and Wellbeing, North East, Public Health England, Newcastle upon Tyne, UK; School of Architecture, Planning & Landscape, Newcastle University, Newcastle upon Tyne, UK; Fuse, The Centre for Translational Research in Public Health, Newcastle upon Tyne, UK

**Keywords:** planning, takeaways, COVID-19, public health, food environment

## Abstract

**Aims:**

Planning regulations have been used to prevent the over-proliferation of hot food takeaways, minimising the impact of local obesogenic environments. To help mitigate the effects of lockdown, the UK government introduced temporary changes in March 2020 to Planning Regulations for England, allowing food retailers to open for takeaway services beyond ‘ancillary’ level without needing to apply for planning permission through permitted development rights (PDR). Businesses are required to notify their local authority (LA) when they implement PDRs. To better understand the impact of regulations on the policy and practice of key professional groups, Public Health England commissioned Teesside University to undertake scoping research in the North East of England.

**Methods:**

A focus group and interviews were conducted with 15 professionals from 7 of 12 North East LAs. Professions included Planners, Public Health Leads, Environmental Health Officers and Town Centre Managers. Data were analysed using a codebook thematic analysis approach. An interpretation meeting with some participants was conducted.

**Results:**

LAs were not aware of most businesses notifying them of new regulation adherence despite taking up PDRs, but were considered low-priority with many lacking formal recording procedures. There were concerns about health consequences of the changes, and consensus relating to ongoing issues with capacity across all professional groups, largely due to the continuing pandemic and absence of a strategy out of temporary measures. Concerns existed around ensuring cessation of restaurants trading as takeaways, and hygiene inspections backlog. Many (personally) saw new takeaways as a lifeline, offering broader menus and preserving local economies.

**Conclusion:**

Lack of information around the number of restaurants/pubs using PDR to trade as takeaway services, ongoing capacity issues of LAs and, at the time, the absence of a strategy post regulation changes, meant there were high levels of uncertainty regarding the impacts of these temporary measures.

## Introduction

Due to COVID-19, on 16 March 2020, people within the UK were urged to refrain from all non-essential contact and travel as a means of prevention of disease spread. As death rates from the disease continued to rise, the country was placed in lockdown and on 23 March, people were allowed only to leave their homes for essential reasons such as shopping and (once a day) exercise.

In response to the outbreak, the Communities Secretary announced that the government would be introducing temporary measures to relax planning regulations; this would allow pubs and restaurants to operate as hot food takeaways in order to support people having to stay at home.^1^

Prior to this, in order for businesses to operate as a hot food takeaway, they were required to formally carry out a change of use (under the Use Classes Order 1987). Relaxing the use class restrictions facilitated the change of use without the need for a planning application. It was stipulated that businesses were *required* to inform their local planning authority of a start and end date to the change in use – however, the form of that notification lack detailed prescription.

Introduction of these permitted development rights meant that businesses were free to switch between classes in order to meet the needs of the public while remaining in operation, albeit in a different capacity, that is, a restaurant could operate as a takeaway without planning permission. These rights were initially introduced for a period of 12 months (until March 2021).

There is substantive evidence of the wider link between urban planning and health.^2^ It is also widely acknowledged that the food environment has a role in influencing obesity levels. Internationally, planning regulations have been used to manage food outlets, with a particular focus on restricting the over-proliferation of hot food takeaways and encouraging healthier food environments.^3^ There has been a particular focus on the ‘toxic high-street’ effect, especially in areas of deprivation with high numbers of hot food takeaways, increased litter and anti-social behaviour.^4^ A recent study reported that both Planners and Public Health professionals perceived that planning policies could be used to tackle this issue around the proliferation of hot food takeaways to improve the health of local populations.^5^

In England the National Planning Policy Framework (NPPF)^6^ sets out the government’s planning policies and how they expect them to be applied. The NPPF makes a number of policy statements relating to health, and in particular food access. For example, paragraph 91c states that planning policies and decisions should ‘enable and support healthy lifestyles, especially where this would address identified local health and well-being needs – for example access to healthier food’. Permitted developments are a general planning permission granted by government – unlike most planning permissions which are issued by local authorities (LAs). The principles underpinning permitted development is enshrined in national legislation – The Town and Country Planning (General Permitted Development) (England) Order 2015. The order is a ‘statutory instrument’ therefore it may be amended without parliament having to pass a new Act; and the 2015 order has indeed had many amendments since it was first issued, including those in 2020 that are the subject of this article.

In 2018, approximately 50% of local government areas in England had planning policies in place specifically targeting takeaway food outlets, and 34% of these had a health focus.^7^ The most frequent health-related method applied is exclusion zones around places children and families visit frequently, such as schools.^7^ The other common method applied is to limit the opening of new takeaway food outlets where numbers exceed previously established acceptable thresholds.^7^ These thresholds can take different forms, such as limiting the number of takeaway outlets in consecutive succession, capping the proportion of fast-food outlets within a recognised retail space or restricting additional outlets where local childhood obesity rates are above a predefined ceiling.^7,8^ Despite some of these measures having been in place for a decade along with other obesity prevention interventions, childhood obesity across England has continued to increase, with children living in the most deprived areas being more than twice as likely to be obese than those living in areas of least deprivation.^9^

One possible explanation for the lack of progress in obesity rates is the large reductions in Local Government funding since 2010, potentially leading to a reduced provision of health-promoting public services. Funding reductions have been the greatest in more deprived areas, with these areas seeing the worst changes in life expectancy, increasing the gap between the most and least deprived quintiles by 3% to 4%.^10^

### Deprivation and the North East of England

The North East of England has long-term socio-economic challenges that developed throughout the 20th century and worsened in the wake of the deindustrialisation of the 1970s and 1980s, making it among the poorest regions in the UK. Like other poorer regions, the North East has tended to recover slowly from economic downturns which in turn have tended to widen long-run socio-economic disparities.^11^ In this context, geographic health disparities have worsened over the past decade and the region has, for example, the lowest life expectancy at birth in England.^12^

As well as being more likely to have a shorter lifespan, those based in the North East spend a greater proportion of their lives in poor health, and are more prone to dying prematurely through preventable diseases.^13^ The North of England includes roughly 50% of the poorest neighbourhoods despite only representing 30% of the population,^12^ with the gap continuing to widen throughout the past five governments.^13,14^ This inequality has equated to an estimated 1.5 million additional premature deaths since 1965.^15^

These inequalities were driven further by COVID-19. During the first wave, mortality rates were significantly higher, with 12.4 more people per 100,000 dying in the Northern Powerhouse cities.^16^ Mortality rates in care homes that were attributable to COVID-19 were higher in the North than the rest of England over a 1-year period, and there were 10% more occupied hospital beds by COVID patients in the North compared with the rest of England.^17^ The UK economy also lost up to £5 billion in reduced productivity due to reductions in mental wellbeing across the North.^15^ This disparity in productivity is expected to worsen for successive generations unless an adequate COVID-19 recovery strategy is implemented,^15^ leading to a consensus among practice professionals and academics that research concerning the structural determinants of poverty should be prioritised to reduce health inequalities.^18^

There has been well-documented pressure on LAs as a result of the COVID-19 pandemic. The central government has provided some support but has said there is no blanket guarantee, with the National Audit Office reporting that the large funding gaps and low levels of reserves mean some authorities are at risk of financial failure.^19^

### COVID-19 response and changes to planning

Although temporary changes introduced in March 2020 allowed businesses to trade beyond the ‘ancillary’ level,^20^ there was still a requirement to notify LAs of such changes. However, despite these regulations, conversations nationally with LA colleagues suggested that, in practice, this did not occur.

A regulatory impact assessment was not completed before the policy was implemented, and the explanatory memo accompanying the regulations stated that monitoring and evaluation would be unnecessary given the short-term nature of the regulations.^21^ The explanatory memos both for the original temporary regulations and for when the policy was extended to 2022, do not identify the socio-economic and health impacts of the changes.^21,22^ The only impact identified states ‘The amendment in relation to the temporary provision of takeaway food will result in a benefit to owners of permitted venues as they will not need to apply to the local planning authority for this temporary use’ with no mention of an impact assessment apart from the procedurally related.^22^

Regulatory functions on the planning system are devolved to Scotland, Wales, and Northern Ireland. Although no new planning regulations were introduced, Scotland and Northern Ireland have taken similar stances to England via various letters to LAs encouraging them to take proportionate enforcement action given the exceptional circumstances of the pandemic. Wales did not state anything specific regarding pub/restaurants trading as takeaways, although they have granted greater flexibility regarding deliveries (see Table 1).

**Table 1:** Responses across UK regions

UK region	COVID-19 takeaway response	Date
England	‘Restaurants and cafes, drinking establishments and drinking establishments with expanded food provision to temporarily provide takeaway food’.^20^	24/03/20
Scotland	‘… Scottish Government consider that, as a matter of urgency, planning authorities should not seek to undertake planning enforcement action which would result in unnecessarily restricting public houses and restaurants providing takeaway services on a temporary basis during the current exceptional circumstances’.^23^	19/03/20
Northern Ireland	‘provide vital flexibility to public houses, restaurants and cafes to keep operating and will ensure people are able to safely stay at home while still supporting local businesses’.^24^	19/03/20
Wales	‘There are supermarkets, food retailers and distribution centres that are subject to planning conditions which restrict night-time and early morning deliveries … The likely pressures on driver capacity mean additional flexibility is needed so retailers can accept deliveries throughout the day and night where necessary’.^25^	13/03/20

As recent as July 2021, it seemed that the Ministry of Housing Communities and Local Government (MHCLG)^i^ was considering whether these changes should be permanent (see quote below), despite the incompatibility with public health NPPF objectives (as previously outlined) to promote healthier food consumption and a healthier weight. A recent speech by the then Secretary of State Robert Jenrick (Robert Jenrick – 6 July 2021 – Local Government Association’s annual conference 2021) had hinted that these changes may be made permanent.^26^ However, in an MHCLG and Ministry of Defence consultation document (5 September 2021), it was made clear that the temporary measures will not be extended.^27^ Food outlets will be able to ‘continue to operate a takeaway service as ancillary to their main business in the absence of this right’.

Surveys have indicated more people accessing takeaways during lockdowns, particularly as restaurants/pubs were not allowed to be open for on-site consumption throughout the changes in regulation.^28^ There are concerns that this increased consumption in takeaway foods will become a habitual long-term shift in behaviour, especially if the temporary regulations are made permanent. These worries are exacerbated given the increased risk of complications from COVID-19 to people who are living with overweight and obesity.

Initially focusing on the North East of England (later aspects expanded to include all of England), the aims of this research were to explore the policy, practice and health implications of the implementation of these measures with key professional groups within the context of Public Health England’s (PHE’s) recently updated childhood obesity plans (July 2020), where the environment and its impact on health has been recognised as complex and multifaceted.

## Method

Ethical approval was sought and granted from a Teesside University Ethics Committee (Ref: 2021 Feb 2740 Moore). Using a purposeful sampling strategy, we sought to recruit LA Public Health, Environmental Health, Planners and Town Centre Manager professionals from across the 12 North East LAs. Potential participants were contacted using professional networks (including RTPI and ATCM), word-of-mouth recruitment in authorities and direct emails sent using publicly available contact details. A focus group and six interviews were conducted between January and March 2021 (during the third National COVID-19 lockdown). The focus group with public health professionals was attached to the end of a standing meeting where all North East LAs are invited. All participants at the meeting were invited to attend the focus group. There was representation from 7 out of 12 LAs who were in attendance on that date.

In total we recruited 15 professionals including Planners, Public Health team members, Environmental Health Officers and Town Centre Managers, from across 7 of 12 North East LAs. Focus groups and interviews were undertaken by members of the research team (A.A.L., C.L.O.M., N.G., and C.B.), held online via Microsoft Teams, and lasted between 30–60 min. We tried several times to arrange additional focus groups, but it was understandably difficult due to these professionals being seconded to Covid-related work during the third national lockdown. Verbal consent was obtained, along with permission to record, prior to any conversations taking place.

Research questions used to direct conversation were co-developed with PHE colleagues (National and Regional) to understand the wider health implications of the new amendment, including how and where the new regulations are being used, plus the number of pubs and restaurants now intending to operate as takeaways.

The questions (below) focused on the perceptions and scale of the temporary changes to planning regulations on the hot food takeaway environment in LA areas within the North East of England.

1. What are the perceived consequences of these temporary regulations on existing national and local planning approaches to managing the hot food takeaway environment?
2. What are the perceived consequences of these temporary regulations on existing national and local priorities on tackling obesity through managing the hot food takeaway environment?
3. What data exist on the take-up of the new regulations and are businesses reporting these changes to LAs?

A survey, using similar questions to those asked in both the focus group and interviews (hosted at onlinesurveys.ac.uk) was sent to all 12 LAs in the North East region and was then widened to all of England to maximise number of respondents. All 12 North East LA websites were also searched systematically for any publicly available information regarding the temporary regulations. Data from the focus group, interviews and survey responses were analysed using a codebook thematic analysis approach.^29^ Descriptive themes were constructed based on similarities identified within the data and refined by means of researcher discussion and consensus.

Following analysis, all participants were invited to an interpretation meeting. This was conducted via Microsoft Teams using Padlet (a digital tool), to present themes, to verify findings and identify any discrepancies or information that may have been overlooked. From here, data were reviewed, and a final set of themes offered, which provide meaning and represent participant experiences and perceptions surrounding the changes in regulations.

## Results

A range of professionals were included in this research from Planners, Public Health team members, Environmental Health Officers and Town Centre Managers.

The perceived consequences of these temporary regulations on existing national and local planning approaches to managing the hot food takeaway environment varied from it having a discernible impact to it having no impact once COVID-19 restrictions were lifted and the temporary measures removed. Regarding the consequences of this temporary measure on tackling obesity, the professionals were concerned that there would be a negative impact on the proliferation of hot food takeaways as a result of this temporary measure and an impact on population rates of obesity. In contrast, others did not perceive there would be an impact. Despite the requirement for businesses to register with LAs, there were little data available on numbers of businesses who had taken up these temporary measures and little evidence of a mechanism by which businesses could register. The results are synthesised around key themes emerging from all the data (focus group, interview, survey, and web-search).

### Professional capacity and notification of business change

There were concerns about the future implications of these measures, as (at the time of the data collection) there was no clear road map for LAs out of these temporary measures. In addition, there was general uncertainty about when professionals would be reverting to their original professional roles following COVID-19 measures (many were working on Covid-related issues). There was concern about increased capacity and resources which would be required to monitor the changes to prepandemic trading operations. Responses suggested there were substantial ongoing issues with capacity across all professions in LAs, heightened by the pandemic. However, this is predominantly due to the response to COVID-19 more broadly, rather than these regulations. Alongside the new regulations were new food environment developments, for example, an increase in takeaways trading from home kitchens. There were concerns regarding the situation post regulations as to who (professional group) is responsible for ensuring restaurants stop their additional trade as takeaways as well as the backlog of food hygiene inspections, as illustrated by the quote below:

*I don’t know who’s going to go around determining all these businesses that didn’t have takeaway permissions. And, I think the businesses will continue to do it, and I don’t know how planning are going to have the resources to go around finding out who is and who isn’t. It’s very hard to prove ‘cos they were always allowed to do a little bit of takeaway*.

There was consensus through the interviews and focus group that small businesses are benefitting significantly from the temporary changes, given many large businesses already had the capacity via delivery services.

**Figure 1:**
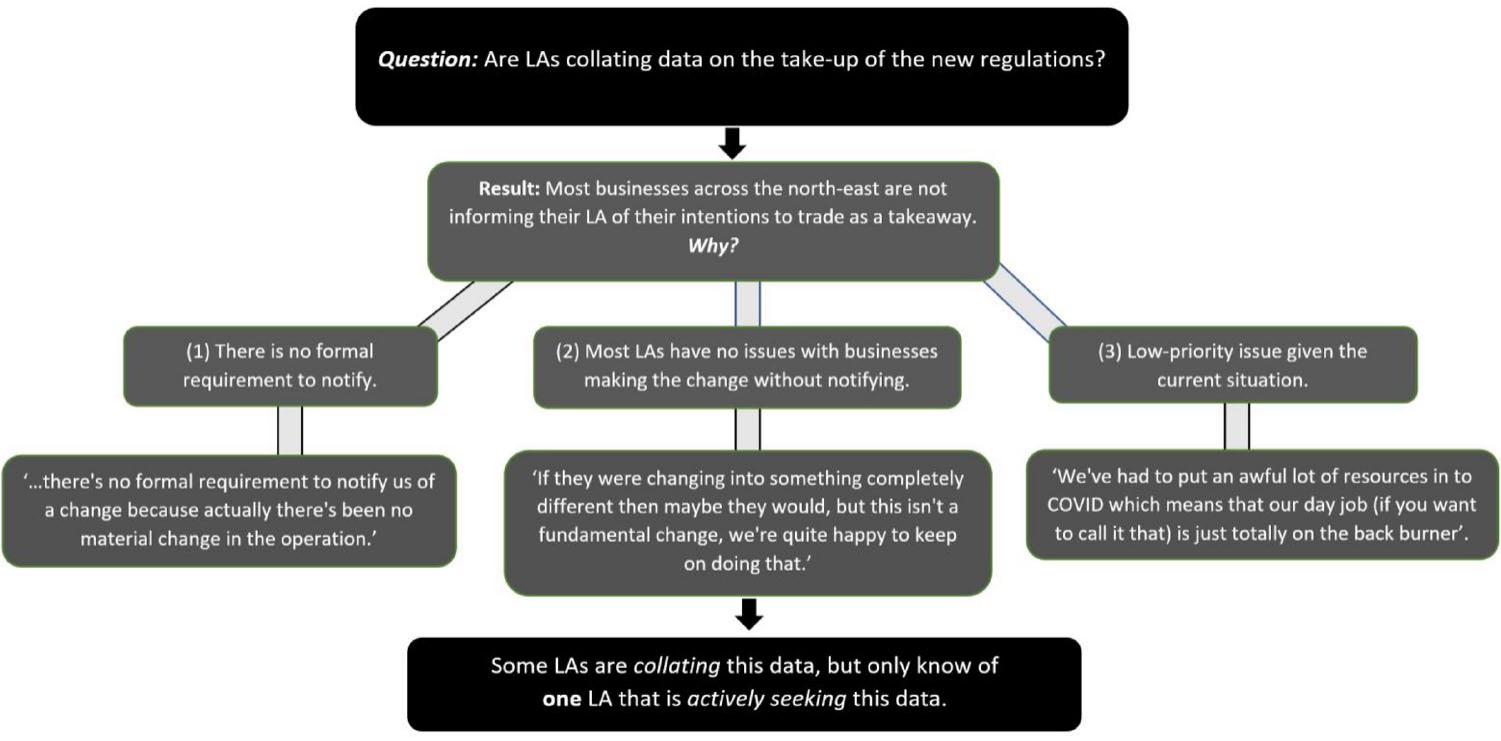
Are local authorities collecting data on the take-up of new regulations?

The conditions of the Amendments to the Town and Country Planning (General Permitted Development) (England) Order 2020 included notifying the local planning authority.^20^ Most businesses were not informing their LA of their uptake of the new regulations. There did not appear to be a formal consistent procedure for notifying LAs (e.g. there was a web-based form used by Plymouth LA). In relation to other Covid issues, the notification of uptake of the new regulations was considered a low-priority issue by most LAs and it was unclear who was responsible. Survey results demonstrated that there was no consistency in awareness of whether LAs were recording businesses giving notifications regarding their intentions to operate as a takeaway in light of regulatory changes (2 LAs were aware of such intentions, 2 were not, and the remaining 3 were unsure) (see Figure 1).

Web-searches of the 12 North East LA websites did not indicate a clear procedure for businesses to notify planning departments. It was unclear and there were no clear records of how many LAs are recording the number of businesses who had taken up these temporary measure England wide (small sample) and across the 12 North East LAs.

### Procedural implications

**Figure 2:**
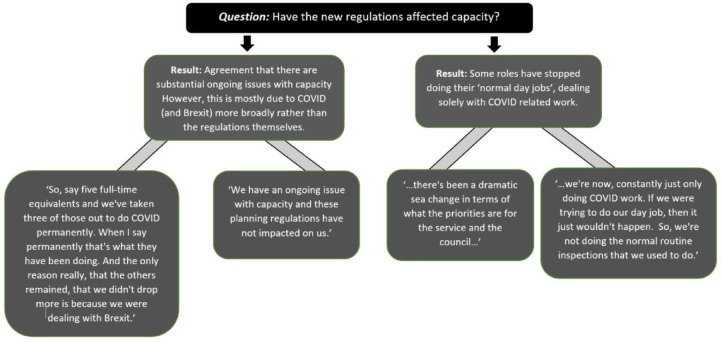
Procedural implications of new regulations

There was a consensus that the COVID-19 pandemic had meant that staff were redeployed within LAs. This was perceived to have had an impact on usual operations, which combined with the absence of a strategy out of these temporary measures (at the time of data collection) led many to raise concerns. These included responsibility for ensuring restaurants stop trading predominantly as takeaways once the regulations revert (March 2022). There was a particular concern around dealing with the backlog of food hygiene inspections. The survey demonstrated that respondents, dependent upon their profession, had differing perspectives on impact of the regulatory changes depending on their role within the LA (Planning, Public Health and Environmental Health Officers). From the focus group, interviews and survey, most businesses across the North East were not informing their LA of their intentions to take up the temporary change in regulations. There were no procedural changes regarding applications for new hot food takeaways. See Figure 2 for further details around the temporary regulations and staff capacity.

### Professional perceptions of hot food takeaways

Professional perceptions of takeaways had not changed (perceived to offer unhealthy, high fat/high sugar/high salt foods). However, on a personal level some respondents described the new takeaway option as a lifeline to local businesses. It was perceived that by offering broader menus and widening consumer choice, community needs could be met, the local economy protected, and the local high street preserved. However, there was an acknowledgement that the COVID-19 pandemic had highlighted changing consumer behaviours in terms of increased takeaway food consumption. There were concerns the increased and broader use of takeaways could become habitual. This could change the local food environment if hospitality businesses, including restaurants, closed as a result of COVID-19 impacts and there is an increased proportion of takeaways. This could to some effect ‘undo’ progress made in reducing hot food takeaways, as evidenced by the below quote:

*it goes against some of the progress we’ve made in terms of trying to reduce the proliferation, …of the hot food takeaways, obviously a little bit different, but you know linking with the SPDs [Supplementary Planning Documents] and so much progress that we’ve made in that area*.

**Figure 3:**
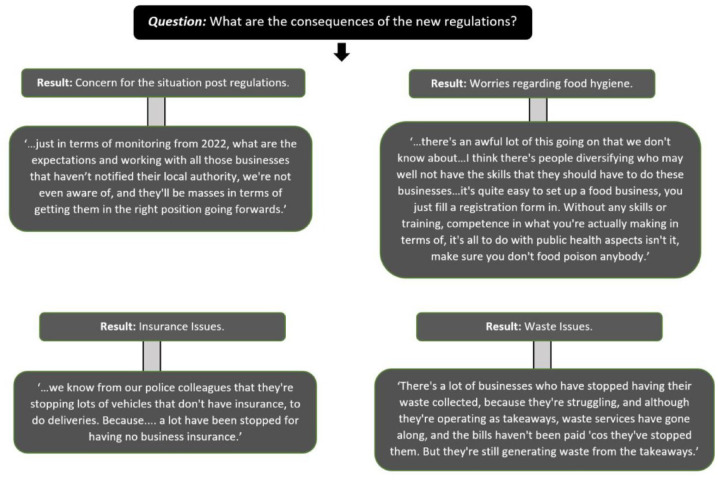
Consequences of the new regulations

Figure 3 highlights other consequences highlighted by respondents, of the temporary measures such as concerns around food hygiene and waste.

**Figure 4:**
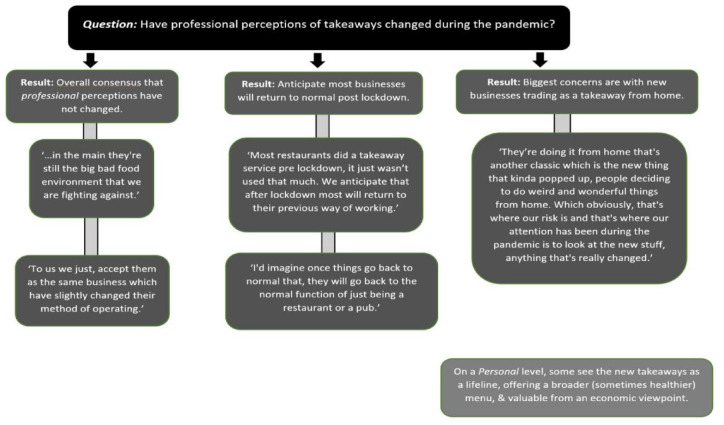
Changes in professional perceptions of takeaways

Within the group of professionals there were mixed opinions regarding consequences on managing the takeaway food environment, health inequalities and obesity; some are concerned about the potential increased prevalence of takeaways, others are not convinced the new regulations will have made a significant difference to consumer behaviour, illustrated by the quote below and further described in Figure 4:

… *in terms of the increase in takeaways and availability, I don’t know if it will … exasperate that kind of health inequality and the divide already in terms of healthier choices in what is available*.

## Discussion

This research suggests that there was a significant impact of COVID-19 crisis on LAs and professionals’ usual roles. This also impacted the professionals dealing with the new temporary changes to Planning Regulations for England allowing food retail (e.g. pubs/restaurants) to be open for takeaway services beyond ‘ancillary’ level. Despite legislation requiring certain hospitality businesses to register their intention to trade as providing additional takeaway services there was little evidence that authorities had set out formal procedures to receive such notifications or had plans to enforce the regulations. There was clear concern that these temporary measures and possible proliferation of hot foot takeaway outlets could become permanent and in turn impact LA public health efforts through the planning systems at a time when LA capacity is clearly stretched.

The recent speech by the then Secretary of State Robert Jenrick (July 2021) clearly implied a move to extend this temporary regulation without any impact assessments being conducted.^26^ However in September 2021, the Ministry of Housing, Communities and Local Government stated these regulations would not be extended beyond March 2022.^27^

This regional research illustrates an on-going tension between LAs’ commitment to health and their serious concerns about their local economies, heightened by the impact of COVID-19. Councils were stretched (financially and staffing capacity) going into the crisis and have not had the capacity to plan beyond it.^19^ On one hand, professionals are concerned about statutory duties including public health and regulating a large backlog of hot food takeaway providers who have been operating for well over a year. On the other hand, simultaneously, Councils are fearful about the long-term impact of the crisis on employment and incomes and an acceleration of difficulties faced on their high streets.

Planning is a long-standing local government responsibility while public health is a relatively new (since 2013) addition to the portfolio. The relationship between Planning departments and Public Health teams differs across LAs and is not always clear. This was evident in the focus group and interviews as well as in previous research.^5,30^ Within Local Government, Councils have discretion over their own internal structures but like many large organisations will usually be made up of several directorates that group together related public service areas. In this context, Planning typically sits alongside Housing, Environmental services, and Economic Development in ‘place’ focused directorates, while Public Health is more likely to be aligned with ‘people’ services such social care.

### Limitations

This research was conducted within a 3-month period (Jan–March 2021) while a third national lockdown was in place due to the COVID-19 pandemic. There are several limitations to the methods. Due to the added pandemic-related pressure, there were issues in liaising and contacting professionals working within LAs. The work was time limited and geographically limited to the North East of England (apart from the national survey). Some findings may not be nationally transferable. There was a low response rate to the national online survey (7 responses nationally) which may be attributed to survey fatigue during the COVID-19 pandemic and there being an ongoing pandemic situation. While this research focuses on professionals, there is ongoing research with consumers in the North East on their perceptions of these temporary changes and eating food prepared outside the home and during the pandemic.

## Conclusion

To help combat the potential economic and social impacts of lockdown in March 2020, temporary regulations were introduced allowing pubs and restaurants to operate beyond ‘ancillary’ level without the need to apply for planning permission. Due to the uncertainty around the number of hot food outlets operating as takeaways, the ongoing LA capacity issues and, until recently, an absence of a clear strategy to exit these temporary regulations, there were high levels of concern among LA professions in the North East. The impact these temporary measures will have on the food environment, influencing food access and population-level public health. There were apprehensions about the implication an increase in the availability of hot food takeaways could have for dietary intake and levels of obesity. There were concerns that these changes will have negative impacts on the work done over the last 10 years to reduce the proliferation of hot food takeaways within the food environment. However, acknowledgements have been made that these temporary measures have enabled small businesses (as well as larger ones) to continue trading under very difficult circumstances and have had a positive impact on local communities. See Box 1 for a summary of research and policy recommendations from this work.

**Box 1.** Key research and policy recommendations from this work

1	Stakeholders considering the need to monitor the uptake of the new regulations versus the impact on the local economy.
2	Consideration of ongoing issues about the revitalisation and diversification of highstreets. This includes the supporting economic recovery which could potentially see an increase in takeaways filling vacant retail properties.
3	It is unclear how many businesses have taken up these new regulations. It would be useful to collate this information England-wide.
4	Propose a clear and transparent plan to move out of these temporary regulations which is driven by local resources, is achievable and aligns with the easing of COVID-19 restrictions.

## Notes

i. As of September 2021, MHCLG has been renamed as the Department for Levelling Up, Housing and Communities (DLUHC).
